# Management and Outcome in 32 Neonates with Thrombotic Events

**DOI:** 10.1155/2011/217564

**Published:** 2011-08-11

**Authors:** H. A. van Elteren, H. S. Veldt, A. B. te Pas, A. A. W. Roest, F. J. Smiers, W. J. Kollen, A. Sramek, F. J. Walther, E. Lopriore

**Affiliations:** ^1^Division of Neonatology, Department of Pediatrics, Leiden University Medical Centre, P.O. Box 9600, 2300 RC Leiden, The Netherlands; ^2^Division of Pediatric Cardiology, Department of Pediatrics, Leiden University Medical Centre, P.O. Box 9600, 2300 RC Leiden, The Netherlands; ^3^Division of Immunology and Hemato-Oncology, Department of Pediatrics, Leiden University Medical Centre, P.O. Box 9600, 2300 RC Leiden, The Netherlands; ^4^Department of Radiology, Leiden University Medical Centre, P.O. Box 9600, 2300 RC Leiden, The Netherlands

## Abstract

*Objective*. To determine the incidence, management, complications, and outcome in neonates with thrombotic events. *Study Design*. We performed a retrospective study of all neonates with thrombotic events admitted to our neonatal intensive care unit from January 2004 to July 2010. *Results*. Thrombotic events were identified in 32 of 4734 neonates (0.7%). Seven neonates were managed expectantly and 25 neonates received anticoagulant treatment. Complete resolution of the clot within 3 months of age was found in 68% (17/25) of the treated and in 86% (6/7) of the nontreated neonates. Major complications due to anticoagulant therapy occurred in 3/25 cases (12%) and included severe hemorrhage (*n* = 2) and abscess at the injection site (*n* = 1). *Conclusion*. Complete or partial clot resolution in neonatal thrombosis occurred in both the treated group and nontreated group. Randomized controlled trials are warranted to determine the optimal management in neonatal thrombosis.

## 1. Introduction

Neonatal thrombotic events are often reported in low birth weight neonates, but little is known about the natural history. Symptomatic thrombosis can lead to life threatening situations such as renal failure, superior vena cava syndrome [1], heart failure [2], and systemic or lung embolism [3]. However, most neonates with thrombosis are asymptomatic and thrombotic events which are often related to indwelling catheters, may be detected by coincidence during routine ultrasound examination [4, 5]. Management options in neonatal thrombosis include a “wait-and-see” policy (expectant management), medical treatment with anticoagulants, or surgery. No randomized trials have been performed in neonates to determine the optimal treatment and consensus management guidelines are not available. Whether medical or surgical treatment is indicated in neonates with thrombotic events remains controversial. The majority of recommendations in the literature are based on uncontrolled studies, small case series, *in vitro* experiments, or extrapolation of study data performed in the adult and pediatric population [6]. 

Despite the limited evidence on the safety and efficacy of anticoagulant treatment in preterm neonates, low molecular weight heparin (LMWH) is increasingly advocated for numerous clinical indications. However, although serious adverse effects associated with anticoagulant treatment are reported to be rare, several major complications have recently been described [7–9]. Data on the safety and efficacy of LMWH in neonates, and in particular in extremely low birth weight (ELBW) neonates, is scarce and based on a handful of case series. The aim of this study is to determine the incidence, management, complications, and outcome in neonates with thrombotic events detected at our centre. 

## 2. Methods

We performed a retrospective chart review of all neonates with thrombotic events detected at our tertiary university neonatal intensive care centre between January 2004 and July 2010. The data on patients included in this study were derived from dedicated database from our neonatology department and pediatric cardiology department. The medical charts of all neonates with thrombotic events were reviewed to determine treatment, complications, and outcome. Ethical approval and informed consent is not needed for this type of study in The Netherlands.

The presence of thrombosis was determined by ultrasonography performed by a radiologist and echocardiography performed by a pediatric cardiologist. Thrombosis was defined as a persistent echodense structure within the heart or a vessel, observed in two dimensions. During the study period, no management guidelines for thrombotic events in neonates were available at our department. Final decision in terms of treatment and management was left to the judgment of the attending neonatologist. 

Criteria for ultrasound examination included persistent thrombocytopenia, ongoing sepsis, or catheter obstruction. 

The following perinatal data were recorded: gestational age at birth, birth weight, gender, sepsis, presence of umbilical catheter (UC) or peripherally inserted central catheter (PICC), thrombocytopenia, number of platelet transfusions, age at onset of thrombotic event, localization of thrombus, neonatal mortality, and complications associated with the thrombotic event and with the management. 

## 3. Results

During the study period, 4734 neonates were admitted to our neonatal intensive care unit (NICU). Thirty-two cases (0.7%) of neonatal thrombosis were identified. The baseline characteristics of the study population are listed in Table 1. The majority were neonates with a gestational age at birth <32 wk (63%, 20/32) or with a very low birth weight (<1500 g) (59%, 19/32). Most neonates (97%, 29/30) had an UC or PICC line prior to or at the time of detection of the thrombotic event. None of the neonates with thrombotic events had signs of renal failure, superior vena cava syndrome, heart failure, or systemic or lung embolism.

Details on the time, size, localization, and management of the thrombotic events are presented in Table 2. The main indication for diagnostic evaluation for thrombotic events was persistent thrombocytopenia (53%, 17/32). Median length and width of the thrombi were 5.3 mm (interquartile range (IQR) 2.1–7.8) and 2.7 mm (IQR 2.0–5.0), respectively. Most thrombotic events were due to intracardiac right atrial thrombi (47%, 15/32).

The majority of neonates (78%, 25/32) were managed with anticoagulant therapy including Tinzaparin (*n* = 20), Nadroparin (*n* = 3), Nadroparin followed by Tinzaparin (*n* = 1), and Nadroparin followed by Phenprocoumon (*n* = 1). 

Complications were found in three cases (12%), all treated with subcutaneous Tinzaparin. In all three cases, anti-FXa levels were within therapeutic range (0.5–1.0 U/mL). One extremely low birth weight (ELBW) neonate developed a severe hematoma at the insuflon (small catheter used for daily injections) injection site, which required emergency surgical drainage. We reported this in a recent case report [9]. Another ELBW neonate developed a large abscess at the insuflon injection site, which required surgical drainage. Lastly, one ELBW neonate developed a grade 2 intraventricular hemorrhage and a unilateral thalamic hemorrhage 4 days after initiation of LMWH. At the time of initiation of LMWH (at 2 weeks of age), no signs of intracranial hemorrhage were detected on (serial) cerebral ultrasound exams. 

Seven cases with neonatal thrombosis were managed expectantly after removal of the UC or PICC line. Followup with serial ultrasound exams showed complete resolution in 6/7 cases (86%) within 2 months. In one case there was only partial resolution of a right atrial thrombus, without clinical relevance (length and width decreased from 2.8 by 1.5 mm to 1.8 by 1.0 mm). One neonate in the “wait-and-see” policy group had a small thrombus in the right atrium and suffered from enterovirus meningitis. The patient died due to systemic and cerebral complications of enterovirus meningitis. Complete clot resolution was observed at follow-up ultrasound before neonatal demise. None of the other patients suffered further complications or died as a result of the thrombus. 

## 4. Discussion

The incidence of thrombotic events in this study was 6.8 per 1000 newborns admitted to our NICU. This rate is almost three times higher than the frequently reported incidence by Schmidt and Andrew of 2.4 per 1000 NICU admissions [10]. Nowak-Gottl et al. reported an incidence of symptomatic thrombosis of 5.1 per 100.000 live births in the period from 1992 to 1994 [11]. However, when admitted neonates with umbilical lines in situ were screened for thrombosis, a much higher incidence rate (from 30% to 43%) was found [12, 13]. Discordant incidences between studies may be due to various methodological differences, including study designs, definitions, and criteria for neonatal thrombosis. The true incidence of thrombosis in our cohort would have been undoubtedly higher if routine ultrasound examinations would have been preformed in all neonates with UC or PICC lines. 

In our study, neonates with thrombotic events were managed either with medical treatment or expectantly, depending on the judgment of the attending neonatologist. The rate of complete clot resolution in our study was high, both in the treatment group and in the expectant management group, without clot-related complications in both groups. Optimal management of neonatal thrombosis is controversial and consensus guidelines are not available. Management options include a “wait-and-see” policy (expectant management), medical treatment with anticoagulants, or surgical treatment. Recent guidelines advise LMWH treatment in neonatal thrombosis with a total duration of 6 weeks to 3 months [6]. However, in this study, we found a high rate of potentially fatal complications in the treatment group. Similar cases of major complications have recently been reported. In a case report, Obaid et al. described an ELBW neonate with a severe hematoma after treatment with LMWH [8]. The neonate developed rapidly a progressive compartment syndrome at the site of Enoxaparin administration with an indwelling catheter and necessitated surgical decompression.

In a retrospective study of 16 neonates (of whom 12 were preterm) treated with LMWH, Malowany et al. reported minor local adverse effects (indurations, bruises, hematomas, or leakage) at the site of the indwelling subcutaneous catheter in 9 (56%) neonates, but also major bleedings in 5 (31%) neonates including scleral haemorrhage, gastrointestinal tract bleeding and infected hematoma [7]. In prospective cohort study of 62 patients, Streif et al. reported four (6%) major bleedings, including two major hematomas at the injection site and one intracerebral hemorrhage 3 days after initiation of therapy [14]. Four other neonates (6%) developed minor bleeding including hematuria, bloody stool, and bleeding at the local administration site. In a retrospective study of 10 preterm neonates treated with LMWH due to venous and arterial thrombosis, Michaels et al. reported bleeding complications in three patients (30%) including mild epistaxis, small subependymal hemorrhage, and oozing at an operative wound site [15]. Finally, in a review article, Malowany reported a 5% incidence of major complications associated with LMWH therapy, based on 240 cases (53 preterm neonates, 61 term neonates, and 126 neonates that were not differentiated on gestational age) [16]. 

Interestingly, all but one major complication occurred even though anti-FXA levels did not exceed therapeutic range which may reflect the difference in pro- and anticoagulant determinants in preterm neonates compared to older children. Unfortunately, pharmacokinetic data on LMWH in preterm neonates is lacking to enhance the urgent need for dose finding studies in preterm neonates. A lower target range may possibly reduce the adverse event range in neonates without compromising clot resolution. 

Alternatively, thrombotic events in neonates may also be managed expectantly. In this study, we found a high rate of complete clot resolution (86%) in the expectant management group. Our findings are in accordance with several reports of spontaneous clot resolution in untreated cases with neonatal thrombosis [17–19]. In a randomized controlled trial comparing long-term and short-term use of umbilical venous catheters in premature neonates, Butler-O'Hara et al. found 24 cases (11%) of thrombosis [17]. Neonates were managed expectantly and clot resolution was reported in all cases without thrombolytic therapy. In a retrospective study, Bendaly et al. describe 19 neonates with intracardiac thrombus of whom 9 were asymptomatic [18]. The majority (8/9) did not receive thrombolytic treatment and resolution was reported in all cases. Lastly, De Godoy et al. reported a newborn with a large cardiac thrombus in the left ventricle causing edema and cardiac dilation [19]. At 55 days, the intracardiac thrombus had disappeared completely without anticoagulant treatment.

In conclusion, our data does not support the routine use of LMWH therapy in neonatal thrombotic events. However, the results of our study should be interpreted with care due to several methodological limitations, including the relative small number of neonates and the retrospective study design. Potential bias associated with retrospective studies does not allow us to compare the outcome between expectant management and medical treatment in our cohort nor the rate of complications. Both LMWH therapy and expectant management may be associated with potential life-threatening complications. Whether treatment should be restricted to a group of neonates with large clots or specific symptoms is not clear. Randomized controlled trials comparing expectant management and treatment with LMWH are urgently needed to determine the optimal management in neonatal thrombosis. 

## Figures and Tables

**Table 1:** Baseline and clinical characteristics of 32 neonates with neonatal thrombosis.

	Treatment group (*n* = 25)	“Wait-and-see” group (*n* = 7)	Total (*n* = 32)
Female gender, *n* (%)	15 (60)	4 (57)	19 (59)
Gestational age (weeks)*	30 (27–38)	28 (25–36)	29 (27–38)
<32, *n *	15	5	20
32 ≪ 37, *n *	2	1	3
>37, *n *	8	1	9
Birthweight (g)*	1256 (817–3300)	938 (734–2735)	1145 (789–3230)
<1000, *n *	8	4	12
1000 ≪ 1500, *n *	6	1	7
>1500, *n *	11	2	13
UC, *n* (%)	18/23 (78)	5 (71)	23/30 (77)
PICC, *n* (%)	14/22 (64)	4 (86)	16/30 (53)
Either UC or PICC, *n* (%)	22/23 (96)	7 (100)	29/30 (97)
Platelet transfusions^a^ *n* (%)	11/21 (52)	2 (29)	13/26 (50)

*Data presented as median (interquartile range (IQR)).

^a^During total hospital stay.

**Table 2:** Characteristics of thrombotic events, management, and outcome.

	Treatment group (*n* = 25)	“Wait-and-see” group (*n* = 7)	Total (*n* = 32)
Age at onset (days)*	11 (6–15)	15 (10–24)	13 (7–16)
Indication for diagnostic evaluation			
Thrombopenia, *n *	14	3	17
Ongoing Sepsis, *n *	4	2	6
Chance finding, *n *	3	1	4
Catheter obstruction, *n *	4	—	4
Thalamusbleeding, *n *	—	1	1
Localization			
Portal vein, *n *	2	—	2
Ductus venosus, *n *	4	1	5
Heart, *n *	10	5	15
Iliac vein, *n *	1	—	1
Basilical vein, *n *	2	1	3
Femoral vein, *n *	4	—	4
Inferior vena cava, *n *	2	—	2
Hepatic Vein, *n *	1	—	1
Duration of treatment (days)*	90 (37–90)	—	—
Follow up ultrasound exam^a^			
Complete resolution, *n* (%)	17 (68)	6 (86)	23 (72)
Partial resolution, *n *	6	1	7
Unchanged, *n *	1	—	1
Unknown, *n *	1	—	1
Major treatment complications, *n *	3	—	3

*Data presented as median (interquartile range (IQR)).

^a^Follow up ultrasound performed within 3 months after detection of neonatal thrombosis.
